# Do socio-demographic factors modify the effect of weather on malaria in Kanungu District, Uganda?

**DOI:** 10.1186/s12936-022-04118-5

**Published:** 2022-03-22

**Authors:** Katarina Ost, Lea Berrang-Ford, Katherine Bishop-Williams, Margot Charette, Sherilee L. Harper, Shuaib Lwasa, Didacus B. Namanya, Yi Huang, Aaron B. Katz, Bwindi Community Hospital, IHACC Research Team, Kristie Ebi

**Affiliations:** 1 School of Epidemiology and Public Health, University of Ottawa, Ottawa, Canada; 2 Priestley International Centre for Climate, University of Leeds, Leeds, UK; 3 School of Interdisciplinary Science, McMaster University, Hamilton, Canada; 4 Department of Geography, McGill University, Montreal, Canada; 5 School of Public Health, University of Alberta, Edmonton, Canada; 6 Department of Geography, Geo-Informatics and Climatic Sciences, School of Forestry, Environmental and Geographical Sciences, College of Agricultural and Environmental Sciences, Makerere University, Kampala, Uganda; 7 Indigenous Health Adaptation To Climate Change, Research Team, Edmonton, Canada; 8 Uganda Martyrs University, Kampala, Uganda; 9 Faculty of Health Sciences, Uganda Martyrs University, Kampala, Uganda; 10 Department of Atmospheric and Ocean Sciences, McGill University, Montreal, Canada; 11 Department of Health Services, University of Washington, Seattle, USA; 12 Bwindi Community Hospital, Kanungu, Uganda; 13 Center for Health and the Global Environment, University of Washington, Seattle, USA

**Keywords:** Malaria, Climate change, Weather, Meteorological, Sex, Age, Indigenous, Batwa, Bakiga, Sociodemographic modifiers, Uganda

## Abstract

**Background:**

There is concern in the international community regarding the influence of climate change on weather variables and seasonality that, in part, determine the rates of malaria. This study examined the role of sociodemographic variables in modifying the association between temperature and malaria in Kanungu District (Southwest Uganda).

**Methods:**

Hospital admissions data from Bwindi Community Hospital were combined with meteorological satellite data from 2011 to 2014. Descriptive statistics were used to describe the distribution of malaria admissions by age, sex, and ethnicity (i.e. Bakiga and Indigenous Batwa). To examine how sociodemographic variables modified the association between temperature and malaria admissions, this study used negative binomial regression stratified by age, sex, and ethnicity, and negative binomial regression models that examined interactions between temperature and age, sex, and ethnicity.

**Results:**

Malaria admission incidence was 1.99 times greater among Batwa than Bakiga in hot temperature quartiles compared to cooler temperature quartiles, and that 6–12 year old children had a higher magnitude of association of malaria admissions with temperature compared to the reference category of 0–5 years old (IRR = 2.07 (1.40, 3.07)).

**Discussion:**

Results indicate that socio-demographic variables may modify the association between temperature and malaria. In some cases, such as age, the weather-malaria association in sub-populations with the highest incidence of malaria in standard models differed from those most sensitive to temperature as found in these stratified models.

**Conclusion:**

The effect modification approach used herein can be used to improve understanding of how changes in weather resulting from climate change might shift social gradients in health.

**Supplementary Information:**

The online version contains supplementary material available at 10.1186/s12936-022-04118-5.

## Background

Malaria continues to pose a threat to human health worldwide. Approximately 92% of all malaria cases in 2017 occurred in the World Health Organization (WHO) African Region. Five of these countries, primarily in sub-Saharan Africa, accounted for half of the malaria cases worldwide [1]. Uganda accounted for 4% of all cases of malaria in 2017, and is among the countries facing significant challenges to *Plasmodium falciparum* malaria elimination due to the presence of highly competent mosquito vectors and lack of infrastructure systems in place to support elimination [1, 2]. Climate change threatens progress made towards malaria elimination in many areas of the world including Uganda. The Intergovernmental Panel on Climate Change (IPCC) concluded with medium to high confidence that climate change could alter the geographic range of the *Anopheles* vector, creating the potential for longer transmission seasons and increasing the number of people at risk, and noted that this projection varies regionally [3].

Among the most vulnerable to the effects of climate change are indigenous people who already face disproportionate burdens of health and social inequity [4]. Sub-Saharan African indigenous people, in particular, often have more health challenges than non-indigenous people living in the same geographic areas [5, 6]. This inequity in health outcomes is frequently rooted in colonization and the social determinants of health, including discrimination, loss of traditional lands, marginalization, and limited access to healthcare services [7, 8].

In recent years, a number of countries have moved to establish nation-wide policies regarding climate change adaptation activities, including in Uganda [9–11]. However, nation-wide policies risk aggregation of diverse at-risk populations, masking important trends at a more granular level. Localized research in vulnerable populations can underpin resource distribution and identification of focal vulnerabilities [12]. The districts in Uganda vary greatly in their geography, demographic makeup, primary health concerns, and in the ways in which they experience climate change impacts. Factors such as sociodemographic makeup of a district will be important in determining the most effective course of adaptive action within specific communities. While many studies have quantified the association between temperature and precipitation for malaria transmission [13–15], these studies typically do not consider how socio-demographic characteristics modify these associations. This study aims to address this gap through evaluation of the role of sociodemographic factors—age, sex, and ethnicity—in modifying the association between temperature and malaria in Kanungu District Uganda from 2011 to 2014 using data from the Bwindi Community Hospital. This study explored effect modification by examining how socio-demographic variables interact and by stratifying models, which has important implications for public health policy under a changing climate.

## Methods

### Kanungu district, Uganda

**Fig. 1:**
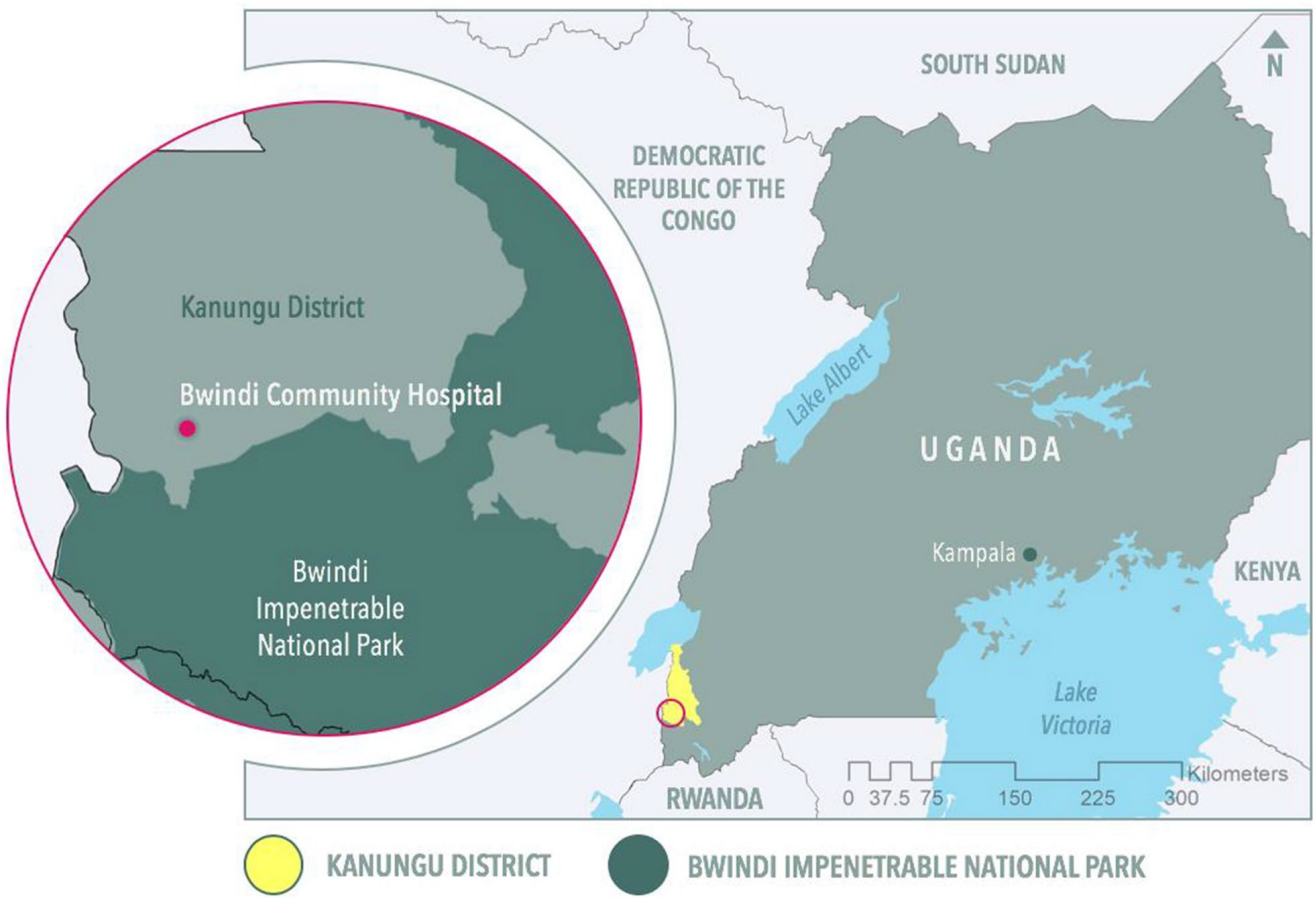
Map of Kanungu district and location of Bwindi community hospital

This study was conducted in Kanungu District, located in southwestern Uganda, near Bwindi Impenetrable National Park (Fig. 1). Over the last 50 years, this region of eastern Africa experienced an increase in seasonal mean temperature [16, 17]. Warming trends are likely to continue, with an increase in mean temperature of up to 2.0 °C projected by 2030, and an increase in regional drying [16].

The region is primarily inhabited by Bakiga and indigenous Batwa people, both of whom face relatively high ill-health burdens when compared with the national average. Both Bakiga and Batwa people are highly vulnerable to health impacts of a warming climate, and have identified malaria, food insecurity, and gastrointestinal illnesses as climate-sensitive health concerns [18, 19]. The indigenous Batwa experience higher prevalence of malaria compared to the Bakiga, 9.4% versus 4.5%, respectively [2]. This difference in malaria prevalence is paralleled by a range of health and socio-economic disparities between the two populations (Table 1), including reduced life expectancy [19]. The Batwa were forcibly removed from their ancestral lands with the creation of the Bwindi Impenetrable Park in 1991 (Fig. 1), where they relied on subsistence hunting and gathering; eviction from the park forced them into settlement in agrarian communities outside the park boundaries (19). There are currently approximately 6700 Batwa individuals living in southwestern Uganda [20], 900 of whom live within Kanungu District. There are no notable ecological or geographic differences in the areas where the Batwa or Bakiga live that would increase risk of malaria in either population (Fig. 1).

**Table 1:** Socio-economic and health differences between Bakiga and Indigenous Batwa populations (adapted from MacVicar et al. 2017a [21])

Health and socio-economic measure	Batwa (proportion of the population (%))	Bakiga (proportion of the population (%))
Malaria prevalence among adult ^a^	6.45	4.46
Moderate acute malnutrition among adult women^b^	45.86	0.42
Household mosquito net use (did not have nets)^c^	70.99	53.56
Access to handwashing facilities (did not have access to handwashing)^d^	73.85	56.40
Access to soap (did not have access to soap)^e^	73.85	56.40

^a^Prevalence of positive malaria antigen detection test in both July 2013 and April 2014—survey of all Batwa adults, sample of Bakiga adults[2]

^b^Classified as moderately malnourished according to the Uganda ministry of health integrated management of acute malnutrition guidelines)[22]

^c^[2]

^d,e^Only asked of people that had access to hand washing facility, for example for the Batwa, 32 or 94% of the households that had access to handwashing had access to soap[2]

The Bwindi Community Hospital (BCH) was founded in 2003 as a clinic to primarily serve Batwa [23]. Since its founding, BCH has expanded into a large facility that includes six in-patient wards, including a pediatric, adult, maternity, and immunodeficiency hospital wards, as well as an out-patient ward, and several satellite clinics for remote settlements [23]. BCH operates on a fee-for service model, and donations help to subsidize an insurance scheme for residents who qualify [18, 24]. All Batwa residents are covered under this insurance plan (eQuality health insurance), which has enrolled 34% of its catchment area in the plan as of 2020 [23, 25].

Kanungu District is a rural area of rolling hills located at an elevation of 1,310 m above sea level [26]. There are four species of malaria parasite that affect humans in Uganda, the most virulent being *P. falciparum* [27, 28]. *Plasmodium falciparum* is the primary endemic malaria parasite found in the Kanungu region and is most often carried by the *Anopheles gambiae* mosquito species [29].

### Data collection

#### Hospital data

Electronic records of patients with a malaria diagnosis from 1-Jan-11 through 21-Dec-14 were obtained through partnership with BCH. Malaria diagnosis was defined as any case with a positive rapid antigen diagnostic test (RDT) or blood slide in conjunction with symptoms. Individual inpatient records from the hospital were merged with insurance coverage data based on patient ID to provide additional data on sex, age, and ethnicity [24]. Data were de-identified prior to analysis to ensure the confidentiality of patients. In total, there were data for 39,287 admissions (all diagnoses) at BCH and 6602 malaria admissions were reported during the years 2011–2014. Of these, 18,846 (48%) of the admissions and 3440 (52%) of malaria admissions, could be matched on sex, age, and ethnicity, and were retained for this study; cases with incomplete information were excluded. Approximately 51% of data were missing information on ethnicity, which was one effect modifier of interest. Excluded cases had a similar demographic distribution to the final sample used in the analysis based on the initial testing (Additional file 1: Table S1).

#### Meteorological data

Meteorological data were estimated from the European Centre for Medium Range Weather Forecasts Re-analysis (ERA)-Interim Climate Database that combined data from multiple sources. The ERA-Interim climate databases have a spatial resolution of 0.75° by 0.75° . Daily values for total precipitation (i.e. rainfall (mm)) as well as maximum, minimum, and average temperature (°C) were obtained for all dates matching the extracted medical records (i.e. 1 January, 2011 to 31 December, 2014) [24]. Meteorological data were merged with the BCH data based on date of admission; lags were then created to account for the assumed time between mosquito/parasite development, point of infection, and finally the day of admission.

The research team focused on the extent to which non-meteorological variables modified the effect of temperature on malaria hospital admission incidence. As such, models did not aim to maximize precision in the specification of the temperature-malaria association, but rather assess the extent to which this generalized association is sensitive to effect modification. Lags were created for both ambient temperature and precipitation, both important predictors of malaria risk [3], out to six months prior to admission date under the a priori assumption that a biologically plausible time lag for malaria would not extend past four months, and would not be less than one month [29–34]. A combined 12 and 13 week lag—the time between admission date and temperature preceding that date by 77–91 days—in mean weekly temperature was identified as having the most significant and strongest association with malaria hospital admission rates and was thus chosen for further analysis (Table 2). The team converted the variable (temperature) into a binary variable reflecting the highest quartile (versus the lowest 3 quartiles combined) of mean weekly temperature 12 and 13 weeks prior to admission in order to evaluate ‘hot’ weeks versus milder or ‘cooler’ weeks. Precipitation was not found to be significant in bivariate analysis, so a binary variable for season was created based on date of admission; rainy seasons were defined as March-June and September–November, and dry seasons were defined as December-February and July–August [35]. Season was retained in all models to account for the dependent nature of temperature and precipitation in the mosquito-weather relationship [13]. Because of the importance of precipitation in this relationship the research team performed an additional sensitivity analysis using rainfall as the predictive variable in the models, and assessed the effect on the size, direction, and significance of the other model coefficients.

**Table 2:** Incidence Rate Ratio (IRR) for malaria hospital admission incidence and temperature, by weekly lag

Lag by week	IRR	p-value	95% CI
10	1.02	0.63	(0.94, 1.11)
11	0.99	0.82	(0.91, 1.08)
**12**^**a**^	**1.09**	**0.04**	**(1.00, 1.19)**
**13**^**b**^	**1.12**	**0.01**	**(1.03, 1.22)**
14	1.06	0.15	(0.98, 1.16)
15	1.07	0.09	(0.98, 1.18)

^a,b^Bold text indicates time lags between admission date and temperature preceding that date selected for use in final models

### Data analysis

#### Conceptual approach

**Fig. 2:**
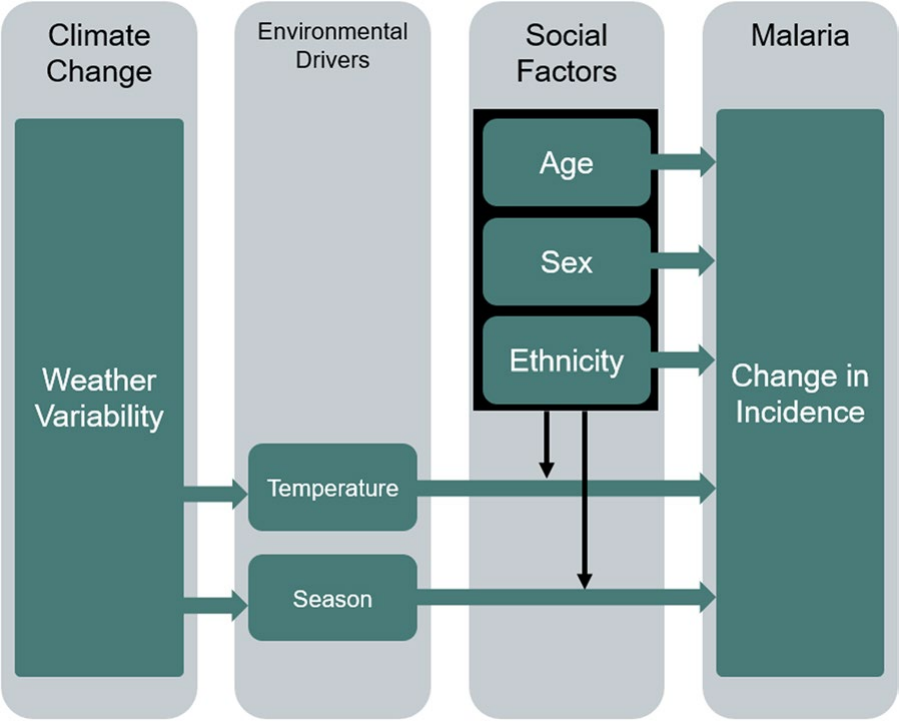
Conceptual model illustrating social modification of malaria-weather relationship: boxes and horizontal arrows represent main pathways of interest in climate–malaria relationship, while the black box represents our sociodemographic variables of interest. Bold vertical arrows indicate theorized effect modification

Effect modification occurs when a variable differentially modifies the observed effect of a risk factor on disease status. This study examined the ways in which age, sex, and ethnicity changed the association of temperature with malaria hospital admission incidence (Fig. 2). Examining effect modification can be achieved through two methods: interaction and stratification. As such, three types of models were used: (1) a baseline model that did not include interaction variables or stratify data; (2) models with weather- and age, sex, and ethnicity interaction variables; and (3) models stratified by age, sex, and ethnicity. The team compared model results among these types of models, comparing the magnitude and direction of the associations between temperature and malaria admissions. Effect modification methods were informed by [24, 36].

### Baseline model

The research team first constructed a model with all meteorological and socio-demographic variables to measure the extent to which temperature is associated with malaria hospital admissions without accounting for any effect modification. We retained sociodemographic variables age, sex, and ethnicity in the model as fixed effects along with year and the meteorological variable season.

### Interaction model

The team used interaction variables to estimate the marginal effects of temperature and age, sex, and ethnicity. This interaction approach took into account interaction and confounding between variables in the model. While theoretically more analytically robust, the coefficients of interaction variables are sensitive to sample size and can be less intuitive to interpret.

### Stratified models

Stratification of models involved fitting separate models for each level of strata. Using stratified models provided a more interpretable estimates of the effects of weather on malaria in different age, sex, and ethnicity strata. This approach is less sensitive to small sample sizes, which was particularly pertinent given the sample size for Batwa in the dataset was small. The team compared incidence rate ratios (IRRs) between demographic variables using a ratio of ratios approach [37].

### Model variables

A summary of variables used in the models is provided in Table 3. The count of weekly malaria hospital admissions was the dependent (outcome) variable of interest. Temperature was the independent (exposure) variable of interest: specifically, the highest quartile (versus the lowest 3 quartiles combined) of mean weekly temperature 12 and 13 weeks prior to admission was examined. Age, sex, and ethnicity were examined as potential effect modifiers. Age was categorized into the following categories: < 5 years, 6–12 years, 13–18 years, 19–55 years, and > 55 years of age. Additionally, the research team conducted a sensitivity analysis on age to verify that results were not sensitive to different age cut-offs. Ethnicity was divided into the two main groups: the Indigenous Batwa, and all other ethnic groups, primarily consisting of ethnic Bakiga. Year and season were identified a priori as confounding variables.

**Table 3:** Description of dependent, independent, effect modification, and control variables used in effect modification analysis

Variable (units)	Description
Dependent (outcome) variable	
Weekly malaria cases	Total case count per 7 day period
Independent (exposure) variable	
Mean weekly temperature ( °C) with a 12–13 weekly average temperature lag	Binary: lower (cooler) quartiles 1–3 (referent); top (hottest) quartile
Effect modification variables	
Ethnicity	Binary: Bakiga and other (referent); Batwa
Sex	Binary: male (referent); female
Age	Categorical: < 5 years (referent); 6-12 years; 13-18 years; 19-55 years; > 55 years
Confounding (control) variables	
Season	Binary: dry (referent); wet *Modelled with an interaction term with the independent variable*
Year	Categorical: 2011 (referent), 2012, 2013, 2014

### Negative binomial multivariable regression models

Researchers used a negative binomial multivariable regression model with the count of total weekly malaria cases as the dependent (outcome) variable. For the population at risk (offset) variable, we used the weekly total admissions to BCH for any diagnosis. Year was controlled for in all models as a fixed effect. All analyses were conducted in STATA v.15.1 (Stata Corp., USA).

#### Baseline model

We first built an unstratified model with no interactions that included sociodemographic variables as fixed effects and meteorological exposures:

$${\text{ln}}\left( {weekly\,malaria\,counts} \right)\,=\,{\beta_0} + {\beta_1}\left( {mean\,weekly\,T^\circ L12 - 13} \right) + {\beta_2}\left( {sex} \right) + {\beta_3}\left( {age} \right) + {\beta_4}\left( {ethinicity} \right) + {\beta_5}\left( {season} \right) + {\beta_6}({\text{Year}}) + \ln \left( {{\text{population}}\,{\text{at}}\,{\text{risk}}} \right)$$

#### Interaction model

The team examined interaction between socio-demographic variables (i.e. age, sex, ethnicity, and season) and temperature, and their association with malaria hospital admissions. To illustrate the size, direction, and confidence interval of interactions, researchers evaluated linear combinations of estimates for weather with season, sex, age, and ethnicity. The team included all interaction variables in a single model. The final model equation used for analyses was:

$$\ln \left( {weekly\,malaria\,counts} \right)\,=\,{\beta_0} + {\beta_1}\left( {mean\,weekly\,T^\circ L12 - 13} \right) + {\beta_2}\left( {sex} \right) + {\beta_3}\left( {age} \right) + {\beta_4}\left( {ethinicity} \right) + {\beta_5}\left( {season} \right) + {\beta_6}\left( {Year} \right) + {\beta_7}\left( {mean\,weekly\,T^\circ L12 - 13\, * \,season} \right) + {\beta_8}\left( {mean\,weekly\,T^\circ L12 - 13\, * \,sex} \right) + {\beta_9}\left( {mean\,weekly\,T^\circ L12 - 13\, * \,age} \right) + {\beta_{10}}\left( {mean\,weekly\,T^\circ L12 - 13\, * \,ethinicity} \right) + {\beta_{11}}\left( {mean\,weekly\,T^\circ L12 - 13\, * \,Year} \right) + \ln \left( {population\,at\,risk} \right)$$

#### Stratified models

The team ran the baseline models stratified by age, sex, ethnicity. Stratified models also contained all control and interaction variables as fixed effects to minimize the effect of confounders in the analysis.

### Ethics

This study approved by ethics boards at McGill University, the University of Guelph, the University of Alberta, and the University of Washington, as well as Bwindi Community Hospital. All personal identifiers were removed from the dataset before analysis. This research is conducted within the broader IHACC project, in partnership with Makerere University, and in collaboration with Batwa health and development programmes in Kampala and in the region. Communities have already consented to ongoing collaboration with the IHACC research project and team. At the time of data collection, the UNSCT was not accepting applications and was not granting ethics approvals.

### Limitations

Data represent a short period of time (less than 4 years) and were, therefore, insufficient to infer relationships between malaria and climate change for long timeframes. Additionally, though wet and dry season were included in the models to represent the complex relationship between meteorological variables like temperature and precipitation, and malaria, there are limitations to excluding precipitation/rainfall as a variable. Future work should examine these meteorological variables in this context in more detail. The Batwa sample in the dataset was small, reducing statistical power in our analysis. Despite this, the team chose to examine ethnicity, given its indicative role as a potentially important driver of vulnerability to malaria in the region and given the Batwa rank malaria as a top climate-sensitive health outcome [2, 19, 38]. In doing so, the research team sought to avoid exclusion of small, but marginalized and high-risk indigenous people from climate-health analytic research [19]. Understanding malaria impacts in view of weather and climate in small vulnerable groups is vital in informing health policy.

## Results

### Descriptive statistics

Of the hospital admissions data with complete sociodemographic records over the study period, the research team found that 56.7 percent of malaria cases in the sample occurred during the wet season from March–June or September–November. Sex was relatively evenly distributed, with 53.1 percent of malaria cases being female, comprising 56.1 percent of the total admissions data. A majority (36.9%) of the total hospital admissions were 19–55 years old, and 35.6 percent of 6–12 year old children who were admitted to the hospital had a malaria infection. Only 238 hospital admissions (1.2% of the sample) were recorded as being Batwa; 22.7% of Batwa hospital admissions were malaria cases during the study period, and 18.2% percent of the Bakiga hospital admissions were malaria cases (Table 4).

**Table 4:** Descriptive statistics of variables included in final models data from Bwindi Community Hospital, Uganda (2011–2014).

Demographics				
	No. of (x) demographic out of all admissions	Proportion of (x) demographic out of all admissions	No. of (x) demographic out of all malaria admissions	Proportion of (x) demographic out of all malaria admissions
Female	10,565	56.10%	1826	53.10%
Male	8281	43.90%	1614	46.90%
0–5 years	5687	30.20%	1143	33.20%
6–12 years	2514	13.30%	896	26.00%
13–18 years	1950	10.30%	414	12.00%
19–55 years	6957	36.90%	895	26.00%
55 + years	1738	9.20%	92	2.70%
Ethnicity (Bakiga)	18,608	98.70%	3,386	98.40%
Ethnicity (Batwa)	238	1.30%	54	1.60%
Season (wet)	10,957	58.14%	1948	56.63%
Season (dry)	7889	41.86%	1492	43.37%
Total number*	18,846		3,440	
Meteorological variables				
Descriptive temperature statistics throughout (x) year (celsius)	Mean	Min	Max	
2011	18.91	13.13	27.51	
2012	19.07	12.22	28.67	
2013	19.54	12.96	28.98	
2014	19.55	13.32	29.19	
Average daily and yearly total rainfall (mm)	Average daily	Yearly total		
2011	3.55	1296		
2012	3.55	1300		
2013	3.22	1174		
2014	3.07	1197		

### Baseline model

The weekly incidence rate of malaria hospital admissions was 1.27 (0.90, 1.80) times higher during weeks with hot weather (highest quartile 29.30–29.42 °C) compared to weeks with cooler weather (three lower quartiles 29.08–29.30 °C) (Table 5). The weekly malaria hospital admissions incidence rate among the indigenous Batwa was 1.08 (0.76, 1.55) times higher than for Bakiga. The weekly incidence rate of malaria hospital admissions for females was 0.91 (0.84, 0.98) times the rate of males. Compared to children 0–5 years old, youth 6–12 years had the highest weekly incidence rate of malaria hospital admissions, followed by youths aged 13–18 years. Weekly malaria hospital admission incidence rates were 1.30 (1.01, 1.68) times higher during the dry season compared to the wet season. Furthermore, the sensitivity analysis using rainfall as the predictive variable in the models suggested very little difference in the models, so it was removed from the analysis.

**Table 5:** Results of baseline model measuring the effect of temperature on malaria with sociodemographic variables as fixed-effects

Model variables	Baseline model IRR (95% CI)	p-value
Temperature ‘Cool’ quartiles	*Referent*	*Referent*
Temperature ‘Hot’ quartile	1.27 (0.90, 1.80)	0.18
Bakiga	*Referent*	*Referent*
Batwa	1.08 (0.76, 1.55)	0.66
Male	*Referent*	*Referent*
Female	**0.91 (0.84, 0.98)**	**0.02**
0–5 years old	*Referent*	*Referent*
6–12 years old	**1.64 (1.48, 1.82)**	** < 0.001**
13–18 years old	1.08 (0.94, 1.24)	0.28
19–55 years old	**0.65 (0.59, 0.71)**	** < 0.001**
55 + years old	**0.28 (0.20, 0.39)**	** < 0.001**
Season (wet)	*Referent*	*Referent*
Season (dry)	**1.30 (1.01, 1.68)**	**0.04**

*Bold indicates a p-value of < 0.05

### Evidence of effect modification of the temperature-malaria association

#### Interaction model

The association of temperature with malaria differed by age, sex, and ethnicity (Table 6). Women experienced a higher weekly incidence rate of malaria hospital admissions compared to men, with this difference substantially higher during hotter lagged weeks (top quartile of mean temperature) compared to cooler lagged weeks. During weeks prior to admission in the three combined cooler quartiles of temperature, the weekly incidence rate of malaria hospital admission between men and women were similar (IRR = 1.02 (0.86, 1.22)). During the hottest weeks before admission, however, the weekly incidence rate of malaria hospital admissions among women was significantly higher (IRR = 2.02 (1.03, 3.09)) than the rate among men. Increases in the weekly incidence rate of malaria hospital admissions were higher in the wet season than the dry season, and higher in the hottest lagged temperature quartile (IRR = 0.45 (0.20, 0.99)) than the cooler lagged quartiles of the dry season (IRR = 0.23 (0.10, 0.51)). Compared to children 0–5 years old, 13–18 year olds had the lowest incidence rate ratio, and the highest being among 55 + year olds. The association between temperature and malaria was higher among the Batwa than the Bakiga, and was more than 50% greater for the Bakiga during the hottest lagged temperature quartile (IRR = 1.63 (0.64, 4.16)). All of these estimates, however, had wide confidence intervals, which could be due to the small Batwa sample size that limits statistical power to detect significant differences.

**Table 6:** Incidence rate ratio (IRR) interaction model results, including sociodemographic variables as effect modifiers

	Interaction model, results by temperature quartile		
	Quartile 1–3 (cool) IRR (95% CI) p-value	Quartile 4 (hot) IRR (95% CI) p-value	IRR hot/IRR cool within strata of ethnicity, sex, age, and season *Ratio of Ratios (ROR)*
Bakiga	*Referent*	*Referent*	*Referent*
Batwa	0.82 (0.34, 1.99) 0.67	1.63 (0.64, 4.16) 0.31	1.99
Male	*Referent*	*Referent*	*Referent*
Female	1.02 (0.86, 1.22) 0.81	**2.02 (1.03, 3.09)** **0.001**	1.98
0–5 years old	*Referent*	*Referent*	*Referent*
6–12 years old	0.96 (0.74, 1.24) 0.75	**1.90 (1.31, 2.74)** **0.001**	1.98
13–18 years old	0.92 (0.64, 1.33) 0.65	**1.82 (1.19, 2.77)** **0.01**	1.98
19–55 years old	0.96 (0.72, 1.29) 0.78	**1.90 (1.32, 2.73)** **0.001**	1.98
55 + years old	1.43 (0.57, 3.59) 0.44	**2.83 (1.05, 7.67)** **0.04**	1.98
Season (wet)	*Referent*	*Referent*	*Referent*
Season (dry)	**0.23 (0.10, 0.51)** ** < 0.001**	**0.45 (0.20, 0.99)** **0.047**	1.96

Interpretation for ethnicity: the Batwa weekly malaria hospital admission incidence rate was 1.63 times the rate of admission for Bakiga during the lagged hot temperatures. The ratio of ratios for Batwa vs Bakiga in the hot quartile over the cool quartiles was 1.99

*Bold indicates a p-value of < 0.05

#### Stratified model

**Fig. 3:**
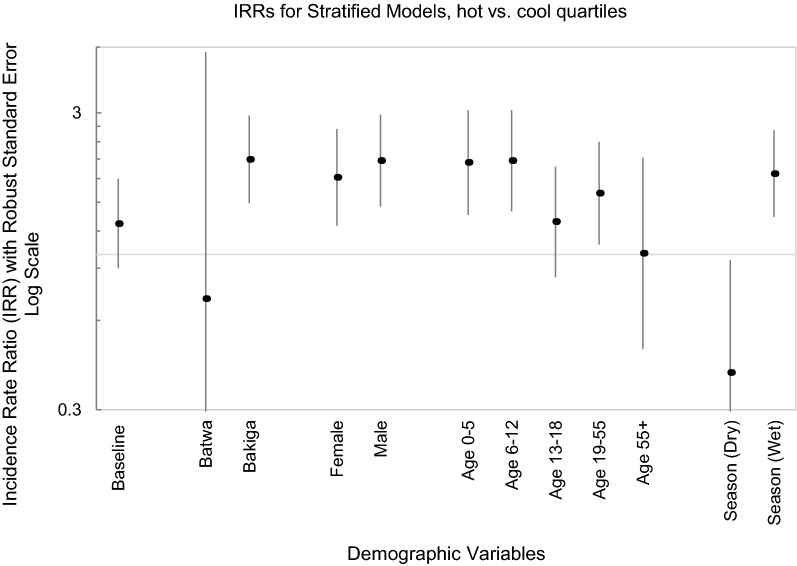
Logarithmic scale; incidence rate ratios by demographic category for stratified models, gridline marks IRR of 1, stratified models for season do not include interaction term for season and temperature

Results differed slightly between interaction and stratified models (Table 7). The Bakiga had a higher association between temperature (hot versus cold quartiles) and malaria incidence compared to Batwa (Batwa IRR = 0.71 (0.10, 4.81) versus Bakiga IRR = 2.09 (1.49, 2.94)). This translates to a ratio of ratios of 0.34, indicating that the increase in weekly malarial hospital incidence rates during the hottest quartile weeks was 0.34 times lower than the rate of the Bakiga. Similar to the interaction models, the results indicated that 6–12 year old children and males had a higher magnitude of association of weekly incidence rate of malaria hospital admissions with temperature compared to other age categories and females, respectively. There was an overall increase in weekly malaria hospital admission incidence rates during the wet season during times of high (4th quartile) temperatures (Fig. 3).

**Table 7:** Incidence rate ratio (IRR) stratification model results with sociodemographic variables as effect modifiers

	Models stratified by social factor; IRR (95% CI)										
	Ethnicity		Sex		Age (years)					Season	
	Bakiga	Batwa	Male	Female	0–5	6–12	13–18	19–55	55 +	Season (wet)	Season (dry)
Temperature quartile 1–3	*Referent*	*Referent*	*Referent*	*Referent*	*Referent*	*Referent*	*Referent*	*Referent*	*Referent*	*Referent*	*Referent*
Temperature quartile 4 p-value	**2.09 (1.49, 2.94)** ** < 0.001**	0.71 (0.10, 4.81) 0.72	**2.07 (1.45, 2.96)** ** < 0.001**	**1.82 (1.25, 2.65)** **0.002**	**2.04 (1.36, 3.06)** **0.001**	**2.07 (1.40, 3.07)** ** < 0.001**	1.29 (0.84, 1.98) 0.25	**1.61 (1.08, 2.40)** **0.02**	1.01 (0.48, 2.12) 0.99	**1.87 (1.34, 2.62)** ** < 0.001**	**0.40 (0.17, 0.96)** **0.04**
IRR strata1/ IRR strata0	*Referent*	0.34	*Referent*	0.88	*Referent*	1.01	0.63	0.79	0.50	*Referent*	0.21

Interpretation for ethnicity: the Bakiga weekly malaria hospital admission incidence was 2.09 times greater during weeks in the hottest temperature quartile than in the coolest quartiles, compared to Batwa, who had 0.71 times greater incidence in weeks in the hottest quartile. The ratio of ratios (ROR) for Batwa vs. Bakiga in the hot season only was 0.34, indicating that the indicative ‘effect’ of the hottest quartile on malaria incidence was 0.34 times the rate in the Batwa than Bakiga, or that Bakiga incidence was more sensitive to temperature compared to Batwa incidence

*Bold indicates a p-value of < 0.05, **Stratified model for season does not include season-temperature interaction term

## Discussion

This study investigated whether social factors, such as age, sex, and ethnicity, modified the association between temperature and malaria hospital admission incidence. Results indicated that the social variables examined in our models do modify this association, although this modification was not significant or lacked sufficient statistical power to achieve statistical significance in all cases. Although subject to wide confidence intervals, the findings point to the strongest associations between temperature and malaria incidence among 6–12 year olds, the elderly, and indigenous Batwa.

The results for ethnicity differed between the baseline and interaction model results with regards to the magnitude of the association, both of which showing that the Indigenous Batwa have a higher incidence of malaria than the Bakiga. Both the baseline and interaction results differed in the direction of the association between the stratified model which suggested that the indigenous Batwa had a lower incidence of malaria in the hot, compared to cold, temperature quartiles, whereas the Bakiga had a greater incidence of malaria in the hot, compared to cold temperature, quartile. Furthermore, descriptive statistics indicated that 18.2% of all Bakiga hospital admissions were for malaria, and 22.7% of all Batwa admissions were for malaria. The baseline and interaction model results were also consistent with community survey research conducted by Donnelly et al. [2], who found the burden of malaria to be substantially higher among the Batwa than Bakiga. These findings, therefore, illustrate the importance of evaluating data for effect modification to capture how weather impacts malaria differentially for Batwa and Bakiga.

Results for age also differed between the baseline and effect modification models. The baseline and stratified results indicated that the highest incidence of malaria hospital admissions was among 6–12 year old children, which is similar to the established literature on malaria, who found that 0–5 year olds have the highest burden on a global scale [1]; however, in the study interaction model results, the team found that individuals over the age of 55 had a higher incidence rate of malaria hospital admissions in the highest temperature quartile when compared with the referent category of 0–5 year olds. The variation between the established literature on highest risk malaria age groups and the baseline and stratification results in this study could possibly be explained by findings from local community surveys, which found that while 0–5 year old children had higher rates of malaria, this age group was more likely to sleep under an ITN at night than any other age group, suggesting that while malaria rates are high overall for those < 5years, seasonal fluctuations in infection may be moderated by ITN protection [39]. Children 6–12 years old, in contrast, were less likely to have ITN protection [39], and may thus experience wider fluctuations in infection risk associated with temperature, which is consistent with our interaction model results.

Our results cannot be directly used to make conclusions about climate change and malaria due to the short study period. Climate-health projections cannot be inferred from weather and temperature associations. These study results indicated that the association between temperature and malaria was stronger among particular social-demographic strata in the Kanungu District region. Notably, this interaction and/or stratification approach implied that sub-populations with the highest incidence rates of malaria will not necessarily be the same as those with the strongest associations between meteorological variability and malaria incidence. The insights from understanding the causal reasons for these differences can point to how malaria risks might shift differentially across sub-populations under climate change. For example, if climate change acts to magnify and/or extend the number of hot weeks, researchers could speculate that these changes could increase malaria incidence more rapidly among age categories who are unprotected by ITNs at night. A traditional approach to projecting climate risk would assume that since children < 5 years have the highest current rates of malaria according to the literature, emphasis on that population is the highest priority. This effect modification approach suggests that while protecting children < 5 years remains a priority, malaria incidence among children 6–12 years and the elderly may be more sensitive to warming, meriting intervention to prevent increased incidence in those age groups. Similarly, while research previously highlighted higher incidence of malaria among the Indigenous Batwa compared to their Bakiga neighbours, these interaction results suggest that Batwa face the additional burden of higher sensitivity to temperature when compared to the Bakiga. Research by Clark et al. [39] highlighted very low retention of ITNs following free distribution, indicating that Batwa may lack ITN protection during peak infection conditions, and also be less likely to benefit from ITN-distribution interventions. These results point to the particularly high vulnerability of Batwa in a changing climate, with existing high burdens of malaria compounded by higher weather sensitivity and lower uptake of interventions compared to neighbouring Bakiga.

Uganda has several national level calls for stronger climate policy including: the Lake Victoria Basin Report 2018, Uganda’s National Adaptation Program of Action (NAPA) 2007, The Uganda National Climate Change Policy 2015, and a National Policy for Disaster Preparedness and Management 2010 [9, 10, 17]. Most of these policies have broad goals that address national level concerns such as water, agriculture, economic, and preparedness adaptation. In Uganda’s more remote districts, such as Kanungu District, interaction results suggest that while the entire population is more susceptible to malaria compared to the national average, some, like the Batwa and youth, experience higher rates of malaria hospital admissions during periods of high temperatures, and may need additional planning and resource allocation, such as assistance with the removal of mosquito breeding sites around the home, or distribution of mosquito bed nets to achieve more equitable adaptation. Currently Uganda policy prioritizes the distribution of mosquito nets to pregnant women or households with children under the age of 5 [39].

## Conclusion

The effect modification approach used herein can be used to improve understanding of how changes in weather resulting from climate change might shift social gradients in health. These study findings suggest that local level policy may be beneficial in addressing some of the more ‘micro’ level concerns that Ugandan Districts will face, such as differential risk of malaria infection among sub- populations. Local policy could expand to include the Batwa population, youth, and the elderly in their high priority prevention efforts, and prioritize follow-up and retention programming among Batwa.

## Supplementary Information

**Additional file1: Table S1.** Descriptive statistics of variables from the original (full dataset) including variables with missing demographic information from Bwindi Community Hospital, Uganda (2011–2014).

## Data Availability

Due to the nature of this research, participants of this study did not agree for their data to be shared publicly, so supporting data is not available.
