# Correction to: Do socio-demographic factors modify the effect of weather on malaria in Kanungu District, Uganda?

**DOI:** 10.1186/s12936-022-04145-2

**Published:** 2022-04-08

**Authors:** Katarina Ost, Lea Berrang-Ford, Katherine Bishop-Williams, Margot Charette, Sherilee L. Harper, Shuaib Lwasa, Didacus B. Namanya, Yi Huang, Aaron B. Katz, Bwindi Community Hospital, IHACC Research Team, Kristie Ebi

**Affiliations:** 1 School of Epidemiology and Public Health, University of Ottawa, Ottawa, Canada; 2 Priestley International Centre for Climate, University of Leeds, Leeds, UK; 3 School of Interdisciplinary Science, McMaster University, Hamilton, Canada; 4 Department of Geography, McGill University, Montreal, Canada; 5 School of Public Health, University of Alberta, Edmonton, Canada; 6 Department of Geography, Geo-Informatics and Climatic Sciences, School of Forestry, Environmental and Geographical Sciences, College of Agricultural and Environmental Sciences, Makerere University, Kampala, Uganda; 7 Indigenous Health Adaptation To Climate Change, Research Team, Edmonton, Canada; 8 Uganda Martyrs University, Kampala, Uganda; 9 Faculty of Health Sciences, Uganda Martyrs University, Kampala, Uganda; 10 Department of Atmospheric and Ocean Sciences, McGill University, Montreal, Canada; 11 Department of Health Services, University of Washington, Seattle, USA; 12 Bwindi Community Hospital, Kanungu, Uganda; 13 Center for Health and the Global Environment, University of Washington, Seattle, USA

## Correction to: Malaria Journal 21:98 (2022) https://doi.org/10.1186/s12936-022-04118-5

Following publication of the original article [1], the authors flagged that the article had published with several formatting errors: First, the author Kristie Ebi had been erroneously affiliated with all of the affiliations rather than affiliation 13, the correct affiliation; Second, Fig. 3 and Tables 6 and 7 had been incorrectly positioned in the PDF version of the article; and finally, red annotations had been erroneously retained in Additional file 1: Table S1.

The article has now been corrected. The publisher thanks you for reading and apologizes for any inconvenience caused.
