# Supplementary material for: Do socio-demographic factors modify the effect of weather on malaria in Kanungu District, Uganda?
Source: Malar J. 2022 Mar 22;21:98. doi: 10.1186/s12936-022-04118-5 (PMC8939205; doi:10.1186/s12936-022-04118-5)
Supplement: Supplementary file 1 — Additional file1: Table S1. Descriptive statistics of variables from the original (full dataset) including variables with missing demographic information from Bwindi Community Hospital, Uganda (2011–2014). [file 12936_2022_4118_MOESM1_ESM.pdf]

Table S1: Descriptive statistics of variables from the original (full dataset) including variables with missing demographic information from Bwindi Community Hospital, Uganda (2011-2014)

Demographics						
	**All admissions (% total)**		**% demographic recorded**	**Malaria admissions (% total malaria)**		**% demographic recorded**
Female	14,260	(36.30)	54.28%	2,602	(39.41)	51.07%
Male	12,011	(30.57)	45.72%	2,493	(37.76)	48.93%
Missing Sex Information	13,016	(33.13)		1,507	(22.83)	
**Total Sex Reported**	26,271	(66.87)		5,095	(77.17)	
Age 0	8,496	(21.63)	21.89%	1,648	(24.96)	25.48%
Age 1	4,816	(12.26)	12.41%	1,535	(23.25)	23.74%
Age 2	4,269	(10.87)	11.00%	824	(12.48)	12.74%
Age 3	16,739	(42.61)	43.12%	2,160	(32.72)	33.40%
Age 4	4,500	(11.45)	11.59%	300	(4.54)	4.64%
Missing Age Information	467	(1.19)		135	(2.04)	
**Total Age Reported**	38,820	(98.81)		6,467	(97.96)	
Batwa	245	(0.62)	1.26%	56	(0.85)	1.56%
Bakiga	19,158	(48.76)	98.74%	3,541	(53.64)	98.44%
Missing Ethnicity Information	19,884	(50.61)		3,005	(45.52)	
**Total Ethnicity Reported**	19,403	(49.39)		3,597	(54.48)	
Season (Wet)	22,886	(58.25)	58.25%	3,760	(56.95)	56.95%
Season (Dry)	16,401	(41.75)	41.75%	2,842	(43.05)	43.05%
Missing Season Information	-	(0.00)		-	(0.00)	
**Total Season**	39,287			6,602		
**Grand total**	**39,287**			**6,602**		
